# Search Strategy for Search Performance; Off-the-cuff or Being Sensitive

**DOI:** 10.29252/beat-070417

**Published:** 2019-10

**Authors:** Behnaz Rastegarfar, Ali Ardalan, Saharnaz Nejat, Abbasali Abbasali, Mohammad Javad Moradian

**Affiliations:** 1 *Department of Disaster Public Health, School of Public Health, Tehran University of Medical Sciences, Tehran, Iran *; 2 *Department of Epidemiology and Biostatistics, School of Public Health, Tehran University of Medical Sciences, Tehran, Iran *; 3 *Department of Health Sciences Education Development, School of Public Health, Tehran University of Medical Sciences, Tehran, Iran *; 4 *Trauma Research Center, Shahid Rajaee (Emtiaz) Trauma Hospital, Shiraz University of Medical Sciences, Shiraz, Iran*; 5 *Department of Disaster and Emergency Health, School of Management and Medical Informatics, Shiraz University of Medical Sciences, Shiraz, Iran*

**Keywords:** Search strategy, Search performance, PRISMA

***Dear Editor,***

We appreciate the interest of the authors in our article entitled “A productive proposed search syntax for health disaster preparedness research”. They have rightly emphasized on the standard reporting of systematic reviews. However, as it is clear from the title and objective of the published article, we did not report results of a systematic review, our article instead aimed to present a syntax validation process which guide with creating a proper search strategy for systematic reviews on disaster preparedness [1-4]. As such neither a PRISMA flow nor an appraising tools were needed. Importance of having a proper search strategy for systematic reviews through a validation process has been widely mentioned in the literature [5-9]. This is crucial for optimizing the methodology of the systematic reviews and quality of the results accordingly. In regard with inclusion of non-English articles, we believed that the wealth of knowledge that exist in such articles should not have been missed. So we included them using the same eligibility criteria that were applied for English articles. Hand searching is a part of the validation process. We have selected the label “relative recall” to refer to the sensitivity index for the proposed syntax [10-12]. 

Our study presents a base search syntax with relative recall of 0.6 which can help researchers interested on disaster preparedness with finding as many as eligible studies possible [13]. It also helps the researchers to be more specific in case they need to focus on any specific hazard or alternative spelling/combination [1].
